# Surveys of postpartum depression in Miyagi, Japan, after the Great East Japan Earthquake

**DOI:** 10.1007/s00737-014-0459-y

**Published:** 2014-09-10

**Authors:** Hidekazu Nishigori, Junichi Sugawara, Taku Obara, Toshie Nishigori, Kineko Sato, Takashi Sugiyama, Kunihiro Okamura, Nobuo Yaegashi

**Affiliations:** 1 Department of Obstetrics and Gynecology, Tohoku University Graduate School of Medicine, Sendai, Japan; 2 Tohoku Medical Megabank Organization, Tohoku University, Sendai, Japan; 3 Department of Health Sciences, Tohoku University Graduate School of Medicine, Sendai, Japan; 4 Tohoku Kohsai Hospital, Sendai, Japan; 5 Division of Feto-Maternal Medical Science, Tohoku Medical Megabank Organization, Tohoku University, 2-1 Seiryocho, Aobaku, Sendai, Miyagi 980-8573 Japan

**Keywords:** Great East Japan Earthquake, Tsunami, Postpartum depression, Edinburgh Postnatal Depression Scale (EPDS)

## Abstract

This study explores the correlation between the impact of the Great East Japan Earthquake and the incidence of postpartum depression in Miyagi prefecture, Japan. The design used was a cross-sectional study with self-administered questionnaires, 6–9 months after the disaster. The results showed the prevalence of postnatal women with Edinburgh Postnatal Depression Scale (EPDS) score of ≥9 to be 21.3 %. Multivariate analysis showed that exposure to tsunami (odds ratio, 1.80; 95 % confidence interval, 1.16–2.78) was significantly and independently associated with an EPDS score of ≥9. Postnatal women and their children should be treated as a vulnerable population, and a protective framework must be established to prepare for future devastating disasters.

## Introduction

Miyagi prefecture is located on the eastern coast of Japan. Following the Great East Japan Earthquake and Tsunami on March 11, 2011, approximately 12,000 people died or went missing, and more than 460,000 houses and buildings were completely or partially destroyed. Previous studies have addressed that maternal mental health (such as perinatal depression) can be influenced by the devastation caused by a natural disaster (Harville et al. 2009). Perinatal depression affects postnatal women’s health and may impact not only the newborn infant’s quality of care but also the subsequent growth and development of the women’s children. In the present study, we examined the Edinburgh Postnatal Depression Scale (EPDS) for postpartum depression and its risk factors (Cox et al. 1987) to assess the Great East Japan Earthquake’s influence on perinatal women’s mental health in Miyagi prefecture.

## Materials and method

### Study subjects

The study design was cross sectional. Participants were recruited from 15 hospitals and 11 clinics in the coastal area of Miyagi prefecture. They delivered between February 1, 2011, and October 31, 2011, and their homes were destroyed by the tsunami. From September 1 to November 30, 2011, the study’s explanatory leaflet and the agreement document were delivered by mail to 3539 postpartum women. The self-administered questionnaires went to 683 participants, who had agreed with the study, and 677 questionnaires were returned. The Ethics Committee of the Tohoku University Graduate School of Medicine approved this study on June 27, 2011 (Number 2011-103).

### Data collection

We collected the data from the self-administered questionnaires. The cutoff score of EPDS among the Japanese population is 9, which is considered to represent a significant risk factor for postpartum depression (Okano et al. 1996).

### Analysis

Both the percentage of women with an EPDS score of ≥9 and the univariate analysis of their backgrounds were analyzed. Student’s *t* test, the chi-square test, and Fisher’s test were used where appropriate for statistical analysis.

Multivariate logistic regression analyses were performed after adjusting for variables significantly associated with an EPDS score of ≥9 in a univariate analysis and the traditional risk factors for postpartum depression, including age, primiparous status, obstetric complications during pregnancy, employment, and death of loved ones. Adjusted odds ratios (ORs) and 95 % confidence intervals (CIs) were calculated to estimate the risk of an EPDS score of ≥9. All statistical analyses were performed using SAS ver. 9.3 statistical software (SAS Institute Inc., Cary, NC, USA).

## Results

**Fig. 1:**
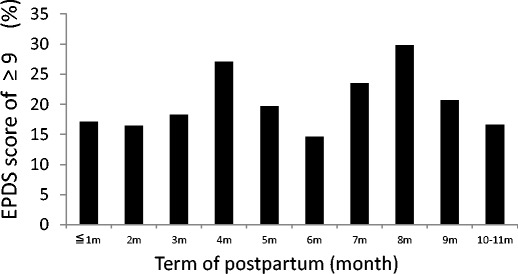
The prevalence of postpartum women with an EPDS score of ≥9 in each month after delivery (*n* = 633). Participants who delivered between February 1, 2011 and October 31, 2011. The study questionnaires were returned from September 1, 2011 to November 30, 2011

The questionnaires were returned from 677 participants, of which 633 were eligible for analysis. The prevalence of postpartum women with an EPDS score of ≥9 was 21.3 %. Figure 1 shows the prevalence of postpartum women with an EPDS score of ≥9 each month after delivery. Univariate analysis showed that age below 25 years (*P* = 0.0153), obstetric complications during pregnancy (*P* = 0.03), baby’s birth weight under 2500 g (*P* = 0.0117), destruction of the home (*P* = 0.0335), and exposure to the tsunami (*P* = 0.0016) were significantly different between women with an EPDS score of ≥9 and those with a score of <9. Multivariate analysis showed that age below 25 years (OR, 2.539; 95 % CI, 1.15–5.60), baby’s birth weight under 2500 g (OR, 2.28; 95 % CI, 1.27–4.09), and exposure to the tsunami (OR, 1.80; 95 % CI, 1.16–2.78) were significantly and independently associated with an EPDS score of ≥9 (Table 1).

**Table 1:** Multivariate logistic regression analyses for postpartum women with an EPDS score of ≥9

	Odd ratio	95 % confidence interval
Age		
≥35 years	1.00	
30–34 years	1.213	0.725–2.031
25–29 years	1.723	0.994–2.987
< 25 years	2.539	1.151–5.599
Multipara (primipara = 0)	1.371	0.848–2.217
No employment (employment = 0)	1.464	0.978–2.192
Postpartum 6 ~ 11 months (≤5 month = 0)	1.161	0.776–1.736
Birth weight ≤2500 g (>2500 g = 0)	2.278	1.269–4.091
Temporary dwelling or refuge (own house = 0)	1.486	0.686–3.217
Exposure to the tsunami (no exposure = 0)	1.795	1.157–2.784

Adjusted by postpartum depression, including age, primiparous status, obstetric complications during pregnancy, employment, and death of loved ones

## Discussion

In this study, the prevalence of postnatal women with an EPDS score of ≥9 was 21.3 %. A previous large population study on Japanese women with postnatal depression reported that 13.9 % of women had an EPDS score of ≥9 (Suzumiya et al. 2004). Our results indicated that postnatal women in Miyagi prefecture’s coastal area had a remarkably higher prevalence of EPDS score of ≥9 after the disaster.

As demonstrated in Fig. 1, the prevalence of a high-risk group for postpartum depression did not exhibit any correlations with the time interval after delivery. These results might suggest that postpartum women have been under chronically stressful conditions even 6 months after the disaster. Further investigations are needed to clarify the precise factors correlated to their situations.

With regard to the risk factors for perinatal depression related to the disaster, previous studies found that exposure to the storm (Xiong et al. 2010), loss of resources (Ehrlich et al. 2010), high earthquake exposure (Qu et al. 2012), and anxiety about earthquakes (Hibino et al. 2009) were more likely to cause depression in pregnant and postnatal women affected by natural disasters. In the present study, we found that exposure to the tsunami was a significant risk factor for postnatal depression. The tsunami disaster was totally unexpected for this vulnerable population; therefore, psychological trauma was much more severe than in previous natural disasters. Interventions of medical and mental care should be carried out immediately to prevent deterioration of maternal pathologic conditions and to observe newborns’ development closely.

## Study limitations

This study has some limitations. It was a cross-sectional study with possible self-report bias, so determining causal relationships was not possible. The prevalence of participants was low, so there was bias toward convenience sampling, and the results may not be applicable to all perinatal women in Miyagi prefecture’s disaster-affected communities.

## Conclusions

In conclusion, the prevalence of postnatal women with an Edinburgh Postnatal Depression Scale (EPDS) score of ≥9 was 21.3 % around 6 months after the disaster. Exposure to the tsunami was more likely to cause postnatal depression in postnatal women. Postnatal women and their children should be treated as a vulnerable population, and a further protective framework is necessary to establish preparedness for future devastating disasters.
